# Atrial Cardiomyopathy: A “Distinct Clinical Entity” for a Deeper Understanding of Atrial Fibrillation and Cardioembolic Stroke

**DOI:** 10.3390/jcm14238363

**Published:** 2025-11-25

**Authors:** Cristian Martignani, Alberto Spadotto, Maria Carelli, Giulia Massaro, Lorenzo Bartoli, Igor Diemberger, Mauro Biffi, Cristiana Corsi, Barbara Zanuttigh

**Affiliations:** 1 Cardiology Unit, Cardio-Thoracic and Vascular Department, Azienda Ospedaliera Universitaria di Bologna, IRCCS Policlinico S. Orsola, 40138 Bologna, Italy; 2 Department of Electrical, Electronic and Information Engineering “Guglielmo Marconi” (DEI), University of Bologna, Campus of Cesena, 40126 Cesena, Italy; 3 Dipartimento di Ingegneria Civile, Chimica, Ambientale e dei Materiali (DICAM), Università di Bologna, 40136 Bologna, Italy

**Keywords:** atrial cardiomyopathy, atrial fibrillation, Embolic Stroke of Undetermined Source, atrial failure, left atrial strain, Cardiac Magnetic Resonance, atrial fibrosis, stroke prevention

## Abstract

A significant portion of embolic strokes occurs without documented atrial fibrillation (AF), challenging the traditional paradigm of cardioembolism. This review addresses the emerging concept of “atrial cardiopathy” as a distinct clinical entity—an underlying atrial substrate abnormality, characterized by fibrosis and dysfunction, that promotes thromboembolism independent of AF. We posit that AF is often a late-stage manifestation of atrial cardiopathy, not the sole trigger for thrombosis. This paper synthesizes the growing evidence linking biomarkers of atrial cardiopathy to Embolic Stroke of Undetermined Source (ESUS). This new framework has profound clinical implications, suggesting a shift from arrhythmia detection to assessing atrial substrate health for stroke risk stratification. Recognizing atrial cardiopathy is fundamental for developing novel “upstream” therapies, such as targeted anticoagulation, aimed at preventing both AF and its devastating thromboembolic consequences. This review critically evaluates the evidence and translational gaps in the field, synthesizing the emerging role of advanced computational modeling as a key future tool for personalized risk stratification.

## 1. Introduction

The relationship between atrial fibrillation (AF) and ischemic stroke has been a cornerstone of cardiovascular medicine for over half a century. As the most prevalent sustained arrhythmia globally, AF is recognized as a potent risk factor, increasing the likelihood of stroke by approximately five-fold [1,2]. The conventional pathophysiological model attributes this risk to blood stasis, particularly within the left atrial appendage (LAA), caused by the loss of organized atrial contraction, which facilitates thrombus formation [3]. Consequently, clinical practice has been anchored to a strategy of arrhythmia detection followed by oral anticoagulation, a paradigm that has successfully prevented countless strokes [2,4].

However, this “AF-centric” model is facing increasing scrutiny due to significant clinical paradoxes. A substantial proportion of ischemic strokes, estimated at 17–30%, are classified as Embolic Stroke of Undetermined Source (ESUS), where extensive diagnostic workup fails to identify a clear cardioembolic, large-artery, or lacunar cause. This diagnostic enigma suggests that our current framework is incomplete [5,6,7]. Furthermore, data from continuous rhythm monitoring in patients with implanted devices have revealed a perplexing temporal dissociation between AF episodes and stroke events. Many strokes occur during periods of sinus rhythm, and the total arrhythmia burden does not always correlate with the timing of the thromboembolic event. This evidence strongly implies that AF may be an intermittent marker of a more persistent, underlying pathology rather than the direct and singular cause of every thrombus [8].

These observations have catalyzed a paradigm shift, moving the focus from a purely electrophysiological phenomenon (AF) to the health of the underlying myocardial substrate [9]. This review will explore the concept of Atrial Cardiomyopathy (AtCM) as a distinct clinical entity, defined by a spectrum of structural, functional, and electrical abnormalities of the atria that promote a prothrombotic state, irrespective of the cardiac rhythm. We will synthesize the evidence positioning AtCM as the “common soil” from which both AF and a significant portion of ESUS arise. This perspective reframes AF as a late-stage, and sometimes absent, manifestation of a progressive disease process [10]. The recent landmark consensus statement from the Heart Failure Association of the European Society of Cardiology (ESC) has formalized this concept, characterizing AtCM as a progressive disorder that culminates in a clinically defined syndrome of atrial failure [11].

This review will deconstruct the pathophysiology of AtCM, with a focus on atrial fibrosis as the central driver of both electrical and mechanical dysfunction. We incorporate the perspective of Papakonstantinou et al. [12] to contextualize AtCM within a unified framework of AF pathophysiology. We will detail the modern, multi-modal diagnostic toolkit for identifying AtCM, from subtle electrocardiographic markers to advanced imaging techniques that can quantify atrial function and tissue characteristics with unprecedented precision. We will examine its primary clinical manifestations, including its critical role in ESUS and its underappreciated contribution to the syndrome of heart failure with preserved ejection fraction (HFpEF). Finally, we will explore the emerging frontiers of atrial electromechanical and hemodynamic modeling, discussing how alterations in these domains may represent the crucial mechanistic link between a fibrotic substrate and thrombogenesis. This new conceptual framework has profound therapeutic implications, and we will conclude by identifying unresolved research gaps to inform a roadmap for clinical implementation.

## 2. The Paradigm Shift: From Atrial Fibrillation to Atrial Cardiomyopathy

The traditional view of AF as a primary “arrhythmogenic” disease is yielding to a more comprehensive, substrate-based model [11,13]. In this evolving framework, AF is no longer considered the starting point but rather the “tip of the iceberg—a clinically conspicuous and often late manifestation of a chronic, progressive underlying pathology [14]. This perspective is encapsulated by the “common soil hypothesis”, which posits that systemic cardiovascular risk factors create a hostile atrial environment conducive to adverse remodeling. Chronic exposure to stressors such as aging, hypertension, diabetes, obesity, and sleep apnea incites a state of chronic inflammation, oxidative stress, and neurohormonal activation within the atrial myocardium [15].

**Figure 1:**
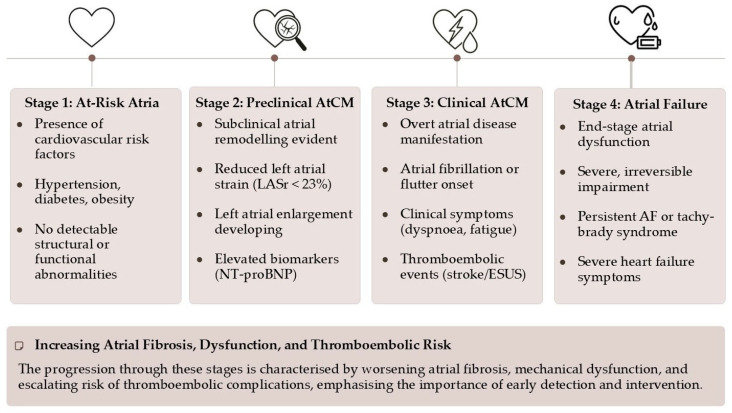
The 2025 ESC/HFA consensus statement classification of atrial cardiomyopathy.

AtCM is best understood as a continuum, a journey from health to failure. The 2025 ESC/HFA consensus statement proposes a practical classification system that delineates this progression (Figure 1) [11]:

1. **Stage 1 (At-Risk Atria):** Characterized by the presence of risk factors without any detectable structural, functional, or biomarker abnormalities. The atria are under stress, but remodeling has not yet begun.
2. **Stage 2 (Preclinical AtCM):** This stage marks the onset of subclinical atrial remodeling. Structural and/or functional abnormalities are now detectable via imaging (e.g., atrial enlargement, reduced strain) or elevated biomarkers (e.g., NT-proBNP), but the patient remains asymptomatic and without a history of atrial arrhythmias or thromboembolism.
3. **Stage 3 (Clinical AtCM):** The disease becomes clinically manifest. This stage is defined by the presence of symptoms (e.g., palpitations, dyspnea, fatigue attributable to atrial dysfunction), documented atrial arrhythmias (AF, atrial flutter), or an atrial-mediated thromboembolic event.
4. **Stage 4 (Atrial Failure):** This represents the end-stage of the disease, characterized by severe and largely irreversible structural and functional atrial derangement. It is defined as “the inability of the atria to guarantee an adequate cardiac output… either at rest or during exercise, in the presence of normal ventricular filling pressures”. This stage is associated with persistent arrhythmias, severe symptoms, and a markedly increased risk of mortality and stroke.

This staged approach has profound clinical implications. It reframes our objective from merely detecting and treating AF to proactively identifying and managing AtCM in its earlier, preclinical stages. Waiting for AF to appear on an ECG is an inherently reactive strategy, intervening only after significant, and potentially irreversible, substrate remodeling has already occurred. The core of this new paradigm is the proactive identification of Stage 2 AtCM, creating a therapeutic window for “upstream” interventions aimed at modifying the underlying substrate, thereby preventing or delaying the progression to clinical arrhythmia and its sequelae [16,17].

While this staged approach provides an essential conceptual framework, its application in routine clinical practice faces several challenges. The thresholds for defining “abnormalities” in Stage 2, for instance, are not universally standardized across different imaging platforms or biomarker assays, creating heterogeneity in diagnosis across studies. Furthermore, the progression is not always linear, and patient-specific factors influencing the transition between stages are still poorly understood. These limitations highlight the need for further research to validate and refine this classification for robust clinical use.

## 3. The Pathophysiological Substrate: Fibrosis as the Central Villain

The histopathological hallmark of AtCM is pathological atrial remodeling, with atrial fibrosis emerging as the central driver of the disease process. Fibrosis, the excessive deposition of extracellular matrix proteins (primarily collagen types I and III), is not a passive process but an active biological response to chronic myocardial injury. It fundamentally disrupts the intricate electromechanical architecture of the atria, leading to a cascade of deleterious consequences [12,14].

Electrically, interstitial fibrosis creates physical barriers that slow and disorganize conduction, leading to electrical heterogeneity and anisotropy [18]. This provides the ideal substrate for re-entrant circuits, which are the fundamental mechanism for initiating and sustaining AF. Mechanically, fibrotic tissue is stiff and non-contractile. Its infiltration into the atrial wall impairs all three phases of atrial function: the reservoir function (passive filling during ventricular systole), the conduit function (passive emptying during early diastole), and the booster pump function (active contraction in late diastole). This mechanical dysfunction leads to elevated atrial pressures, pulmonary congestion, and, critically, blood stasis [19].

The development of atrial fibrosis is a complex process orchestrated by multiple signaling pathways. Chronic hemodynamic stress (pressure or volume overload) and metabolic insults activate atrial fibroblasts into a pro-fibrotic myofibroblast phenotype [20,21,22,23]. Key signaling molecules, such as transforming growth factor-beta 1 (TGF-β1), angiotensin II, and endothelin-1, play a pivotal role in this process. The resulting fibrotic substrate provides the nidus for thromboembolism through mechanisms that perfectly align with Virchow’s triad, adapted to the atrial environment:

- Blood Stasis (Abnormal Flow): Impaired atrial contractility and LAA dysfunction, direct consequences of fibrosis, lead to reduced blood flow velocity and prolonged residence time. This is particularly pronounced within the complex, trabeculated anatomy of the LAA, creating a sanctuary for thrombus formation.
- Endothelial Dysfunction (Abnormal Vessel Wall): The chronic inflammation, oxidative stress, and mechanical stretch associated with AtCM activate the atrial endocardium. This activation leads to a prothrombotic state, characterized by the expression of adhesion molecules and the downregulation of anticoagulant factors like nitric oxide.
- Hypercoagulability (Abnormal Blood Constituents): The systemic inflammatory states that drive AtCM (e.g., diabetes, obesity) also contribute to a systemic hypercoagulable state by increasing levels of circulating pro-thrombotic factors like fibrinogen and plasminogen activator inhibitor-1 [3].

**Figure 2:**
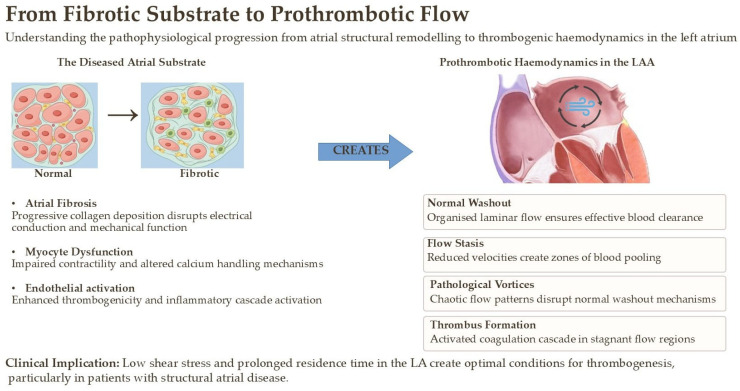
Pathophysiological progression from atrial structural remodeling to thrombogenic hemodynamics in the left atrium.

Therefore, identifying, and ideally quantifying, atrial fibrosis is no longer an abstract pathological exercise; it is a critical step toward understanding a patient’s true risk of both arrhythmia and stroke, independent of the rhythm strip (Figure 2). While fibrosis is recognized as the key driver, its non-invasive quantitative assessment remains a challenge. Integrating multi-parametric imaging with biomarkers and correlating these findings with histological data, as partly explored in large cohorts like MESA, is a critical next step to fully understand the link between the quantity and quality of fibrosis and clinical outcomes [24].

## 4. The Diagnostic Toolkit for Atrial Cardiomyopathy

Recognizing AtCM before the onset of persistent AF requires a modern, multi-modal diagnostic approach that integrates evidence from across the diagnostic spectrum.

### 4.1. Electrocardiographic Clues: Beyond AF Detection

The surface 12-lead ECG, a ubiquitous and inexpensive tool, offers early and powerful clues to the presence of an underlying atrial substrate disease.

- Advanced Interatrial Block (aIAB—Bayes’ Syndrome): Defined as a P-wave duration ≥ 120 ms combined with a biphasic (positive-negative) morphology in the inferior leads (II, III, aVF), aIAB signifies a conduction block in Bachmann’s bundle, forcing a slow, circuitous caudo-cranial activation of the left atrium. It is a potent marker of widespread atrial fibrosis and is strongly associated with incident AF, ischemic stroke, and cognitive decline. Its presence should alert the clinician to a high-risk atrial substrate.
- P-wave Terminal Force in Lead V1 (PTFV1): A classic marker of left atrial abnormality, an abnormally deep and wide terminal negative portion of the P-wave in V1 (≥−0.04 mm·s) reflects the delayed and aberrant depolarization of a diseased left atrium. It has been used as a key inclusion criterion for defining AtCM in major clinical trials investigating ESUS.
- Prolonged P-wave Duration: Even without the specific morphology of aIAB, a simple P-wave duration ≥ 120 ms indicates slowed inter- and intra-atrial conduction and is an independent predictor of incident AF and thromboembolism [25,26,27].

### 4.2. Echocardiographic Innovations: Left Atrial Strain

For decades, echocardiographic assessment of the left atrium was largely confined to anatomical measurements of its diameter or volume. While left atrial enlargement is a robust prognostic marker, it often represents a late and potentially irreversible stage of remodeling [28,29]. The advent of 2D speckle-tracking echocardiography has revolutionized atrial assessment by enabling the measurement of left atrial strain (LAS), a quantitative measure of myocardial deformation [30].

LAS provides a direct window into atrial function. Reservoir strain (LASr), which measures atrial expansion during ventricular systole, has emerged as the most sensitive and clinically relevant parameter. It is a functional barometer of atrial health, reflecting the combined effects of atrial compliance and contractility. A decline in LASr is a hallmark of atrial myopathy, driven by fibrosis and myocyte dysfunction, and critically, it often occurs before significant atrial enlargement. A growing body of evidence demonstrates that reduced LASr is a powerful and independent predictor of incident AF, heart failure hospitalizations, and ischemic stroke in patients in sinus rhythm [31,32,33]. A proposed threshold of LASr < 23% is increasingly recognized as a robust indicator of significant AtCM, earning it the moniker “the troponin of the atrium” for its ability to detect early atrial injury [30].

Notably, data from large prospective cohorts such as the Atherosclerosis Risk in Communities (ARIC) study have robustly demonstrated that LA reservoir strain is a powerful predictor of incident AF and ischemic stroke, outperforming other echo and ECG markers. Crucially, one landmark analysis showed that after adjusting for LA reservoir strain, AF itself was no longer a significant predictor of stroke, cementing the idea that the underlying myopathy, not the arrhythmia, is the primary driver of risk [34].

Despite its prognostic power, the widespread clinical adoption of LA strain faces significant hurdles. These include inter-vendor variability in speckle-tracking algorithms, the need for standardized acquisition protocols, and the lack of universally accepted age- and sex-specific reference values. Furthermore, the optimal strategy for integrating LA strain into existing risk scores, such as the CHA_2_DS_2_-VASc score, remains an area of active investigation and debate [35].

The incremental value of LASr is also evident in various other clinical contexts. In patients with significant mitral regurgitation, it provides crucial insights into the consequences of volume overload on LA structure and function [36]. In aortic stenosis, impaired LASr can predict adverse outcomes and the development of post-TAVR atrial fibrillation [37,38]. Similarly, in hypertrophic cardiomyopathy and cardiac amyloidosis, a reduction in LASr often precedes other signs of clinical deterioration and serves as a powerful early marker for risk stratification [39,40].

### 4.3. Advanced Imaging: Visualizing and Quantifying Fibrosis

Cardiac Magnetic Resonance (CMR) with Late Gadolinium Enhancement (LGE) represents the current gold standard for the non-invasive visualization and quantification of focal atrial fibrosis. Gadolinium-based contrast agents are excluded from healthy myocytes but accumulate in the expanded extracellular space characteristic of fibrotic tissue, appearing as hyperenhanced (bright) regions on LGE images [41].

This technique has transformed atrial fibrosis from a microscopic concept into a macroscopic, measurable therapeutic target. The extent of LGE-detected atrial fibrosis, often classified using the Utah classification (Stage I-IV), has been shown to strongly correlate with the risk of both AF recurrence after ablation and, most importantly, the risk of stroke, independent of the CHADS_2_-VASc score [42]. Patients with extensive fibrosis have a significantly elevated risk of thromboembolism, even without a history of AF. While LGE primarily detects dense, replacement fibrosis, newer CMR techniques like T1 mapping can quantify diffuse interstitial fibrosis, potentially offering an even earlier and more comprehensive assessment of the atrial substrate. LGE-CMR provides the ultimate in vivo confirmation of a diseased substrate and holds promise for guiding patient selection for more aggressive risk-reduction therapies.

While representing a gold standard, atrial LGE-CMR has technical limitations that can impact its clinical utility. The thinness of the atrial wall makes imaging inherently challenging, and there is significant variability in image acquisition protocols and post-processing techniques for fibrosis quantification. The reproducibility of LGE segmentation can be limited, particularly for less dense fibrosis, highlighting a need for standardized methodologies to ensure reliable and comparable results across centers. A summary of these key diagnostic modalities, their parameters, advantages, and limitations is provided in Table 1.

## 5. From Anatomical Imaging to Functional Simulation: The Era of the Digital Twin in Atrial Cardiomyopathy

While advanced imaging techniques like LGE-CMR provide an unprecedented, high-resolution map of the anatomical substrate, their nature remains inherently static. They reveal the “where” of fibrosis but cannot fully elucidate the “how”—how these structural abnormalities translate into dynamic functional deficits. Bridging this gap between static anatomy and dynamic pathophysiology is the frontier of patient-specific computational modeling, a field in which personalized in silico simulations are used to create a “digital twin” of the patient’s atrium. This approach is paramount for understanding the complex interplay between substrate and arrhythmogenesis.

The process begins with the detailed anatomical data derived from CMR or high-resolution CT scans. These images are segmented to create a three-dimensional, patient-specific anatomical mesh of the atria. Crucially, information on the location and extent of fibrotic tissue, as identified by LGE-CMR, can be integrated directly into this model, assigning different material properties to healthy versus diseased tissue regions [43,44]. This anatomically accurate model then becomes the foundation for simulating two critical aspects of atrial function:

1. Electrophysiological Modeling: By applying sophisticated mathematical models of cellular action potentials and electrical propagation (e.g., monodomain or bidomain models), it is possible to simulate the conduction of the electrical impulse across the atrial surface. These simulations can vividly demonstrate how regions of fibrosis slow down conduction velocity, create electrical heterogeneity, and establish the substrate for re-entrant circuits—the very mechanism of AF. This in silico approach allows researchers to test hypotheses about arrhythmogenesis and predict which fibrotic patterns are most likely to sustain AF [45].
2. Biomechanical Modeling: The same patient-specific model can be used to simulate atrial mechanics and contractility. By assigning passive stiffness properties to fibrotic regions and active contractile properties to healthy myocardium, these models can predict regional wall motion, quantify myocardial stress and strain, and compute functional parameters like ejection fraction and reservoir strain. This provides a direct mechanistic link between the extent of fibrosis and the reduction in atrial function observed with echocardiography (LASr), moving from a mere correlation to a cause-and-effect simulation [46,47].

The clinical implications of this “digital twin” approach are profound. Such models hold the potential to move beyond generic risk scores towards a truly personalized risk stratification [48]. They could be used to predict a patient’s likelihood of developing AF, assess their individual stroke risk based on simulated mechanical dysfunction, areas of thrombosis, and even guide therapies by simulating the outcome of ablation and left atrial appendage occlusion procedures before they are performed [49,50,51]. The major challenge remains clinical translation, requiring faster computation and standardized, validated pipelines to ensure reproducibility and clinical utility.

## 6. Clinical Manifestations of Atrial Cardiomyopathy

### 6.1. The Link to Embolic Stroke of Undetermined Source (ESUS)

The concept of AtCM provides the most compelling and unifying pathophysiological explanation for the ESUS paradox [52]. A diseased, fibrotic, and poorly contracting atrium can serve as a thrombogenic nidus even in sustained sinus rhythm. The combination of blood stasis from mechanical dysfunction and an activated endocardium creates a sufficient prothrombotic milieu. In this framework, ESUS is not truly “cryptogenic” but rather a manifestation of undiagnosed or subclinical AtCM [53]. Multiple studies have substantiated this link, demonstrating that biomarkers of AtCM (e.g., elevated NT-proBNP, abnormal P-wave indices, left atrial enlargement, reduced LASr) are significantly more prevalent in patients with ESUS compared to those with other stroke etiologies.

This hypothesis was formally tested in the landmark ARCADIA trial, which randomized ESUS patients with evidence of AtCM (defined by ECG, echo, or biomarker criteria) to either apixaban or aspirin. The trial was stopped for futility, as it did not demonstrate a significant benefit of anticoagulation over antiplatelet therapy in this population [54,55].

However, this neutral result does not invalidate the AtCM hypothesis but rather highlights critical limitations in the trial’s design. The definition of AtCM used for inclusion was broad and non-specific, lacking quantitative imaging validation like LASr or CMR fibrosis. The enrolled population likely included many without advanced substrate disease, diluting the treatment effect. This interpretation is strongly supported by data from the ARIC and MESA cohorts, which demonstrated that when LASr or LGE-quantified fibrosis are included in multivariate models, AF itself ceases to predict stroke independently [24,34]. These findings confirm that structural atrial disease, rather than transient arrhythmia, is the principal embolic driver. Thus, future trials require refined diagnostic enrichment to select true high-risk AtCM patients for targeted anticoagulation [12].

### 6.2. Atrial Failure: The “Missing Diagnosis” in Heart Failure

AtCM is also a critical, yet often overlooked, contributor to the syndrome of Heart Failure with Preserved Ejection Fraction (HFpEF) [56]. Many patients with HFpEF present with debilitating symptoms of dyspnea, exercise intolerance, and fatigue that are traditionally attributed solely to left ventricular diastolic dysfunction. The ESC/HFA consensus statement has championed the concept of Atrial Failure as a distinct clinical syndrome where symptoms are driven, in significant part, by a failing atrium [11].

A stiff, fibrotic atrium cannot relax adequately to accommodate pulmonary venous return (impaired reservoir function) or contract effectively to augment ventricular filling (impaired booster function). Both deficits lead to elevated left atrial pressures, which are transmitted backward to the pulmonary circulation, causing pulmonary congestion and dyspnea [57]. Recognizing the contribution of atrial failure is paramount for accurate diagnosis and management. It forces clinicians to reconsider symptom attribution in HFpEF and opens the door for therapies specifically aimed at improving atrial function and unloading the atrium, representing a new therapeutic frontier in heart failure. Further research is needed to identify specific imaging and circulating biomarkers that can distinguish symptoms driven primarily by atrial failure from those of concomitant ventricular dysfunction, addressing this crucial diagnostic overlap in HfpEF.

## 7. The Hemodynamic Frontier: Flow Dynamics as a Lynchpin in Atrial Thrombogenesis

Once the functional consequences of the fibrotic substrate are understood through electromechanical modeling, the next logical step is to investigate their impact on intra-atrial blood flow. While static anatomical and tissue-characterization techniques like LGE-CMR and echocardiography are invaluable for identifying the presence of a diseased substrate, a complete understanding of thrombogenesis requires a deeper appreciation of its ultimate functional consequence: the derangement of intracardiac blood flow. The complex interplay between abnormal atrial geometry, impaired wall motion, and the resulting blood flow patterns represents a critical, under-explored frontier. Investigating this domain is essential, as hemodynamic forces are the proximate trigger for platelet activation and thrombus formation [58].

The presence of AtCM creates a uniquely prothrombotic hemodynamic environment governed by principles of fluid dynamics. Two key phenomena are central to this process:

- Flow Stasis and Pathological Vorticity: In a healthy atrium, blood flow is organized into smooth, large-scale vortices that facilitate efficient “washout,” particularly within the LAA. In AtCM, non-contractile fibrotic regions and generalized hypokinesis disrupt these physiological flow patterns. This leads to zones of near-stasis, where blood velocity approaches zero, and the formation of abnormal, persistent, small-scale vortices. Within these regions, the residence time of blood elements, including activated platelets and coagulation factors, is dramatically prolonged, increasing the statistical probability of thrombus initiation and propagation. The complex, trabeculated morphology of the LAA makes it exceptionally vulnerable to these phenomena [59].
- Adverse Endocardial Shear Stress: Blood flowing across the endocardium exerts a frictional force known as wall shear stress (WSS). In healthy arteries and cardiac chambers, physiological, laminar WSS promotes endothelial quiescence through mechanotransduction pathways that upregulate antithrombotic factors like nitric oxide. The disturbed flow patterns in AtCM, however, generate pathological WSS profiles, characterized by either abnormally low WSS in regions of stasis or high oscillatory shear stress in areas of turbulent or recirculating flow. It is now well-established that these adverse WSS profiles are potent activators of endothelial pro-inflammatory and pro-thrombotic pathways, such as the NF-κB cascade, leading to a shift toward a thrombogenic endothelial phenotype [60].

The quantification of these hemodynamic derangements represents a critical next step in refining stroke risk stratification beyond conventional metrics. Computational Fluid Dynamics (CFD) has emerged as a powerful, non-invasive tool for this purpose. Approaches range from pure hemodynamic modeling solving 3D Navier–Stokes equations from patient-specific anatomical models, to combined Eulerian–Lagrangian models tracking blood particle trajectories to derive indicators like mean blood age and residence time [61,62]. Other frameworks combine biochemical and hemodynamic modeling, often in 2D, to simulate the spatial distribution of thrombin, platelets, and fibrin [63,64,65].

Integrating these advanced hemodynamic simulations with data on the underlying fibrotic substrate from LGE-CMR could yield highly personalized, multi-scale risk models. Such an approach could identify high-risk individuals who are invisible to current risk scores and would provide a mechanistic basis for selecting patients for advanced therapies, representing a convergence of clinical cardiology, advanced imaging, and biomedical engineering. However, significant translational barriers remain, including the high computational cost and the critical need for robust validation of in silico models against in vivo 4D flow MRI data. Overcoming these challenges will be key to incorporating hemodynamic profiling into personalized stroke risk assessment.

## 8. Therapeutic Implications and Future Directions

The recognition of AtCM as the fundamental pathology necessitates a profound shift in therapeutic strategy—from a reactive, rhythm-focused approach to a proactive, disease-modifying atrial medicine [12].

- Upstream Therapies: The primary goal must be to prevent or reverse adverse atrial remodeling. This begins with aggressive, guideline-directed management of all underlying risk factors, including hypertension, diabetes, obesity, and sleep apnea. Beyond this, several drug classes have shown promise as “upstream” therapies due to their anti-fibrotic and anti-inflammatory properties. Renin–angiotensin–aldosterone system (RAAS) inhibitors have been shown to attenuate atrial fibrosis in experimental models [66]. More recently, SGLT2 inhibitors and GLP-1 receptor agonists have demonstrated remarkable cardiovascular benefits, part of which may be attributable to their favorable effects on atrial structure and function by reducing inflammation and oxidative stress [67,68].
- Rethinking Anticoagulation: The central therapeutic dilemma is whether to anticoagulate patients with severe AtCM in the absence of documented AF. The neutral result of the ARCADIA trial highlights the need for better patient selection. Future trials are imperative and must employ more specific criteria to identify a population with a stroke risk high enough to warrant anticoagulation. This could involve combining biomarkers, for instance, requiring the presence of both severe mechanical dysfunction (e.g., LASr < 20%) and a significant structural substrate abnormality (e.g., severe LA enlargement or extensive fibrosis on LGE-CMR).
- Targeting Fibrosis Directly: As our understanding of the molecular pathways driving fibrosis deepens, novel anti-fibrotic therapies are on the horizon. The development of direct inhibitors of pathways like TGF-β, or agents such as pirfenidone and galectin-3 inhibitors, could one day offer the ability to halt or even reverse the progression of AtCM. In such a future, quantitative imaging with LGE-CMR would be essential not only for diagnosis but also for monitoring the efficacy of these targeted treatments. Future clinical trials of these agents must not only demonstrate efficacy in modifying the atrial substrate but also rigorously evaluate their long-term safety and potential off-target effects.

## 9. Conclusions

The concept of atrial cardiopathy represents a definitive evolution in our understanding of cardioembolic stroke, yet its transition from a research concept to a clinically actionable diagnosis is incomplete. This review has critically summarized the current evidence, highlighting both the promise and the unresolved challenges. While a multi-modal toolkit for identifying AtCM exists, significant knowledge gaps remain. Key among these are the lack of a standardized, quantitative definition for AtCM, the need for prospective validation of imaging thresholds (e.g., LASr cutoffs) in diverse populations, and the limited histological correlation for non-invasive fibrosis imaging.

A clear roadmap for the future is required. This must include: (1) establishing standardized, reproducible methodologies for LA strain and CMR fibrosis quantification; (2) conducting prospective trials, informed by data from cohorts like ARIC and MESA, to define high-risk AtCM phenotypes that warrant anticoagulation; (3) investing in research on novel anti-fibrotic therapies; and (4) validating computational models against robust clinical data to pave the way for personalized medicine. Embracing AtCM is not merely an academic exercise; it is an urgent call to address these gaps and translate our advanced understanding of atrial substrate disease into improved clinical practice and patient outcomes.

## Figures and Tables

**Table 1:** The Diagnostic Toolkit for Atrial Cardiomyopathy.

Modality	Key Parameters and Thresholds	Advantages	Limitations
ECG	P-wave duration: ≥120 ms aIAB (Bayes’): P-wave ≥ 120 ms + biphasic in II, III, aVF PTFV1: ≥−0.04 mm·s	Ubiquitous, low-cost Excellent screening tool Provides physiological data on conduction	Low sensitivity Non-specific Influenced by body habitus
Echocardiography	LA Volume Index: >34 mL/m^2^ LA Reservoir Strain (LASr): <23% E/e’ ratio: >14 (indicates filling pressures)	Widely available, non-invasive Provides both structural and functional data LASr is an early and sensitive marker of dysfunction	Operator-dependent Poor acoustic windows can limit quality LA volume is a late marker
Cardiac MR (CMR)	Late Gadolinium Enhancement (LGE): Detects focal fibrosis (Utah Stage I–IV) T1 mapping/ECV: Quantifies diffuse interstitial fibrosis	Gold standard for tissue characterization Directly visualizes and quantifies fibrosis Highly reproducible	High cost, limited availability Requires contrast agent (contraindicated in severe renal failure) Complex image acquisition/post-processing
